# The Characteristics and Outcomes of Nonhospitalized Patients With Heart Failure in Saudi Arabia: A Contemporary Single-Center Study

**DOI:** 10.7759/cureus.51756

**Published:** 2024-01-06

**Authors:** Ammar G Chaudhary, Shifa J Arshad, Farida W Dahdouleh, Emily L Heaphy, Ioannis E Koulouridis

**Affiliations:** 1 Cardiovascular Diseases Department, King Faisal Specialist Hospital and Research Centre, Jeddah, SAU; 2 Academic and Training Affairs Department, King Faisal Specialist Hospital and Research Centre, Jeddah, SAU; 3 Nursing and Clinical Affairs Department, King Faisal Specialist Hospital and Research Centre, Jeddah, SAU; 4 Research Center, King Faisal Specialist Hospital and Research Centre, Jeddah, SAU

**Keywords:** iron deficiency, mitral regurgitation (mr), dilated cardiomyopathy (dcm), patient outcomes, epidemiology of hf, heart failure

## Abstract

Background

Contemporary data on patients with heart failure (HF) in Saudi Arabia is limited.

Methods

This was a retrospective study of patients who were followed in the HF Clinic at our center after January 1, 2022. The study end date was August 31, 2023. Patients who were alive and followed for <6 months were excluded. We reported the clinical characteristics, utilization of established therapies for HF, proportion of potential candidates for ancillary HF treatments, and rates of HF events and mortality.

Results

A total of 202 patients met the study criteria. The mean age was 56.0 ± 15.2 years. The median follow-up from the initial visit to the study end date was 47 months (interquartile range {IQR}: 29-58 months). Coronary artery disease (CAD) was the cause of HF in 85 (42%) patients. At their latest visit, 103 (51%) patients had diabetes, 82 (41%) were obese, and 134 (66%) received quadruple therapy. Iron deficiency was present in 143 (71%) patients during follow-up. At their latest visit, moderate-to-severe or severe functional mitral regurgitation (MR) and hyperkalemia were present in 15 (7%) and 20 (10%) patients, respectively. The combined annual rate of HF hospitalization and emergency visits for HF was 20%. At least one hospitalization for HF within a year before the study end date occurred in 19 (9%) patients. The annual all-cause mortality was 1.8%.

Conclusion

This contemporary cohort of outpatients with HF was relatively young and had a high prevalence of diabetes, obesity, and iron deficiency. An estimate of potential candidates for iron replacement, transcatheter repair of the mitral valve, novel potassium binders, and the implantation of the pulmonary artery pressure monitor was among the first reported regionally. All-cause mortality was low, yet the burden of HF-related events was significant.

## Introduction

The burden of heart failure (HF) has reached epidemic proportions. According to one estimate, the global prevalence of HF exceeds 64 million, approximating 2/3 of the global prevalence of cancer [1]. Data suggests an increase in the prevalence of HF in Saudi Arabia in the past two decades [2,3]. The clustering of risk factors for HF at an earlier age has been demonstrated in the Saudi population, with the potential to impact the evolution and natural history of HF [4]. These differences in the characteristics of patients with HF in Saudi Arabia were explored in a few reports through datasets that are now a decade old [5,6].

Since the publication of those reports, advancements in therapy for HF have prompted major updates in society guidelines [7,8]. The utilization of this expanded therapeutic landscape is an important quality metric of healthcare delivery and is necessary for resource planning, yet contemporary local data is lacking.

HF is saddled with high rates of morbidity and mortality [7-10]. Outpatients constitute the majority of patients with HF, and those with elevated risk were the source of recruitment in landmark clinical trials [11,12]. However, patients from Saudi Arabia have been underrepresented in such studies, and available registry data on the outcomes of outpatients with HF in Saudi Arabia predated the adoption of newer therapies [5,6].

To address some of these gaps, we sought to report the clinical characteristics, utilization of established therapies for HF, proportion of potential candidates for new ancillary HF treatments, and rates of HF events and mortality among outpatients with HF at our center.

## Materials and methods

Study design

This was a retrospective study of patients who were referred to the HF Clinic at the King Faisal Specialist Hospital and Research Centre, Jeddah, Saudi Arabia. We included consecutive patients of ≥14 years of age who were already followed or newly enrolled in the HF Clinic since January 1, 2022, after the use of sodium-glucose cotransporter-2 inhibitors (SGLT2i) had been fully implemented. The study end date was August 31, 2023. Patients were excluded if they were alive and followed in the HF Clinic for <6 months, had heart transplantation or a left ventricular assist device (LVAD) at the time of referral, or were missing key data necessary to determine the etiology and course of HF.

Patient demographics, the classification and etiology of HF, symptoms, signs, relevant investigations, and treatments were recorded from the initial visit, the latest visit, or both where applicable. The data was derived from electronic clinic records that had been standardized since the inception of the clinic.

Heart failure was defined as a clinical syndrome with symptoms and signs that resulted from any structural or functional impairment of ventricular filling or ejection of blood [7]. A structural abnormality characteristic of HF included left ventricular ejection fraction (LVEF) that was reduced (≤40%) or mildly reduced (41%-49%). In the case of preserved LVEF (≥50%), it referred to the enlargement of the left atrium (left atrial indexed volume of >34 mL/m^2^). The evidence of increased ventricular filling pressure could be any of the following: a) elevated natriuretic peptide (NP) level, such as N-terminal pro-B-type natriuretic peptide (NT-proBNP) of >125 pg/mL; b) an increase in the echocardiographic diastolic parameter E/e’ (≥15) at rest or exercise; or c) pulmonary capillary wedge pressure of ≥15 mm Hg [7]. Patients at risk of HF were considered stage A, those with structural or functional impairment without previous or current clinical features of HF were considered stage B, those with previous or current symptomatic HF were considered stage C, and those with end-stage HF were considered stage D [7]. Iron deficiency was defined as ferritin of <100 ng/mL or ferritin of 100-299 ng/mL and transferrin saturation of <20% [7]. The assessment of LVEF was based on echocardiograms performed at our center. The LVEF at the initial visit was based on the echocardiogram performed within six months prior to the initial visit and, if not available, within one month after the initial visit. The latest LVEF was based on the echocardiogram performed within six months prior to the latest visit, provided that there was at least a six-month interval between the initial and the latest echocardiograms.

Clinical events related to deaths, hospitalizations for HF, emergency department (ED) visits for HF, cardiovascular (CV) hospitalizations unrelated to HF, and non-CV hospitalizations were recorded between the initial visit to the HF Clinic and the end of the study period. All deaths were assessed through available records and/or telephone contact per standard practice. For events other than deaths, only those that occurred at our institution were included.

The study was approved by the Research Ethics Committee of the Office of Research Affairs of the King Faisal Specialist Hospital and Research Centre, Jeddah (KFSH&RC-J) (approval number: 2023-134). The requirement for informed consent was waived because of the retrospective study design.

Statistical analysis

Continuous variables are presented as mean ± standard deviation or median (interquartile range, IQR) and were compared with the paired t‐test, McNemar’s test, or the Wilcoxon signed-rank sum test, according to the distribution of data. Categorical variables are presented using frequencies and percentages and were compared using the chi-square test or Fisher’s exact test. A P-value of 0.05 was considered statistically significant. For outcomes, median follow-up was estimated from the initial visit to the HF Clinic to the study end date. The median follow-up in the HF Clinic was the median of the duration between the initial and latest visits. Data was analyzed using the Statistical Analysis System (SAS Institute Inc., Cary, NC) and the R language and environment for statistical computing (R Foundation for Statistical Computing, Vienna, Austria).

## Results

A total of 219 patients were followed in the HF Clinic between January 1, 2022, and August 31, 2023. After excluding 17 patients (n = 2 post heart transplant, n = 2 post LVAD implantation, n = 12 alive with follow-up of <6 months, and n = 1 with missing data), 202 patients met the study criteria.

General characteristics

Table 1 and Table 2 present the general characteristics and etiology of HF. The mean age was 56.0 ± 15.2 years, and 67 (33%) were females. The median follow-up from the initial visit to the HF Clinic to the study end date was 47 months (IQR: 29-58 months). Heart failure with reduced ejection fraction (HFrEF) was the most common type of HF in 154 (76%) patients. Both HFrEF and HF with improved ejection fraction (EF) (HFimpEF) represented 90% of the cases at the latest visit. Half of the cohort had diabetes mellitus. Obesity was the second leading comorbid condition. Coronary artery disease (CAD) was the cause of HF in less than half of the patients. The remaining patients had multiple etiologies, and 46 (23%) patients had no identifiable cause.

**Table 1:** General characteristics of patients at the latest visit Distribution of variables is expressed in mean ± standard deviation unless stated otherwise. ^a^Median follow-up duration between the initial HF Clinic (HFC) visit until the study end date. ^b^The^ ^classification of heart failure (HF) was determined at the latest visit. HFrEF is HF with reduced ejection fraction (EF), defined as EF of ≤40%. HFmrEF is HF with mildly reduced EF, defined as EF of 41%-49%. HFpEF is HF with preserved EF, defined as EF of ≥50%. HFimpEF is HF with improved EF, defined as EF that was ≤40% and improved by at least 10% to >40%. ^c^The principal cause of HF was based on investigations that were completed by the latest visit per the determination of the managing physician. A mixed cause was considered when ischemic and nonischemic factors contributed to HF. ^d^Comorbid conditions were determined at the latest visit. Moderate chronic kidney disease (CKD) was defined as estimated glomerular filtration rate (eGFR) of <60 mL/minute/1.73 m^2^, and severe CKD was defined as eGFR of <30 mL/minute/1.73 m^2^ IQR: interquartile range

Characteristic (n = 202)	
Age, years	56 ± 15.2
Female gender, number (%)	67 (33.2)
Follow-up, months, median (IQR)^a^	47 (29-58)
Follow-up in the HFC between the initial and the latest visit, months, median (IQR)	42 (24-54)
Region, number (%)	
Jeddah	105 (52.0)
Others	85 (42.1)
Unknown	12 (5.9)
Classification of HF, number (%)^b^	
HFrEF	154 (76.2)
HFmrEF	5 (2.5)
HFimpEF	29 (14.4)
HFpEF	14 (6.9)
Principal cause of HF, number (%)^c^	
Ischemic	85 (42.1)
Nonischemic	113 (55.9)
Mixed	4 (2.0)
Comorbid conditions, number (%)^d^	
Diabetes	103 (51.0)
Hypertension	60 (29.7)
Moderate or severe chronic kidney disease	77 (38.1)
Obesity	83 (41.0)
Stroke	23 (11.4)

**Table 2:** Specific etiology of nonischemic or mixed cause of heart failure (n = 117) ^a^Mixed etiology was determined in four patients: Two patients had autoimmune disease and obstructive coronary artery disease (CAD) and two patients had obstructive CAD with cardiomyopathy that was out of proportion to the degree of CAD. The etiology of cardiomyopathy in the latter two patients could not be determined

Etiology	Number (%)
Viral	3 (2.6)
Autoimmune	7 (6.0)
Peripartum	8 (6.8)
Uremic	5 (4.3)
Chemotherapy	5 (4.3)
Arrhythmia-induced	4 (3.4)
Valvular	6 (5.1)
Stress-induced	3 (2.6)
Hyperthyroidism	1 (0.9)
Hypertension	2 (1.7)
Recreational drug use	3 (2.6)
Muscular dystrophy	1 (0.9)
Congenital heart disease	1 (0.9)
Arrhythmogenic right ventricular dysplasia	1 (0.9)
Hypertrophic cardiomyopathy	1 (0.9)
Restrictive	1 (0.9)
Amyloidosis	2 (1.7)
Familial	7 (6.0)
Heart failure with preserved ejection fraction, garden variety	6 (5.1)
Mixed^a^	4 (3.4)
Unknown	46 (41)

Symptoms and signs

**Figure 1:**
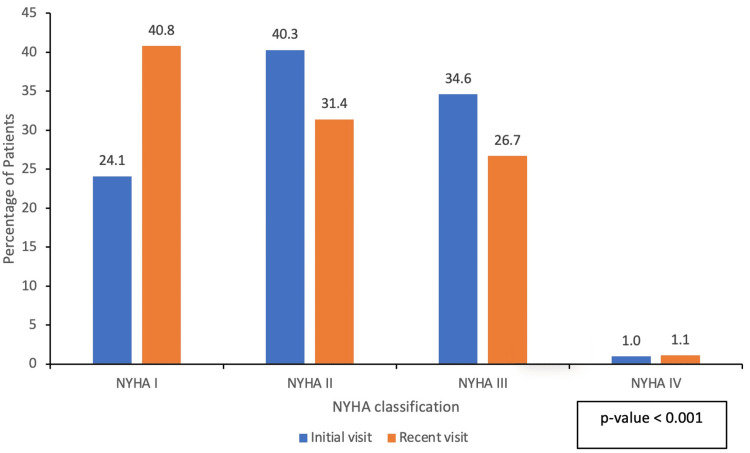
NYHA classification at the initial and latest visit NYHA: New York Heart Association

Stage C HF was the predominant stage of HF (91%) at the initial visit. Dyspnea was reported by 145 (76%) patients on the initial visit versus 113 (59%) patients on the latest visit (P-value < 0.001) (Table 3). The New York Heart Association (NYHA) functional classification improved on follow-up (P-value < 0.001) (Figure 1). Dizziness was the second most common symptom at the latest visit, reported by 83 (41%) patients. Heart rate and systolic blood pressure decreased between the initial and latest visits.

**Table 3:** Clinical features of patients at the initial and/or latest visit, where applicable The distribution of variables is expressed in mean ± standard deviation unless stated otherwise. ^a^Paired observations, n = 191. ^b^Paired observations, n = 195

Clinical feature (n = 202)	Initial visit	Latest visit	P-value
Dyspnea^a^, number (%)	145 (75.9)	113 (59.2)	<0.001
Chest pain, number (%)	-	16 (7.9)	-
Palpitations, number (%)	-	24 (11.9)	-
Dizziness, number (%)	-	83 (41.1)	-
Edema, number (%)	-	12 (5.9)	-
Heart rate, beats/minute^b^	79.1 ± 13.6	76 ± 13.5	0.011
Systolic blood pressure, mm Hg^b^	115.8 ± 18.8	113.2 ± 20	0.067
Weight, kg		78.4 ± 21.0	-
Body mass index, kg/m^2^		29.1 ± 7.2	-

Results of investigations

Table 4 summarizes the results of relevant investigations for HF. The median LVEF increased between the initial and latest visits (27.5% versus 30.0%, P-value < 0.001). Half of the cohort had severe left ventricular (LV) systolic dysfunction at the latest visit. Greater than moderate mitral regurgitation (MR) was present in 15 (7.4%) patients despite the treatment of HF for >6 months.

**Table 4:** Results of investigations at the initial and/or latest visit, where applicable The distribution of variables is expressed in mean ± standard deviation unless stated otherwise. ^a^NT-proBNP is N-terminal pro-B-type natriuretic peptide. Paired observations, n = 192. ^b^Paired observations n = 196. ^c^The lowest simultaneous values for transferrin saturation and ferritin were used. ^d^The estimate is out of n = 197 in whom the status of iron deficiency could be determined IQR, interquartile range; eGFR, estimated glomerular filtration rate

Investigation	Initial visit	Latest visit	P-value
Left ventricular ejection fraction, %, median (IQR)	27.5 (20.0-35.0)	30.0 (22.5-40.0)	<0.001
Left ventricular ejection fraction of <30%, number (%)	112 (55.5)	97 (48.5)	0.061
Moderate-to-severe/severe mitral regurgitation, number (%)	-	15 (7.4)	-
NT-proBNP, pg/mL, median (IQR)^a^	659.5 (297.0-1848.0)	547.5 (215.0-1742.0)	0.609
Peak NT-proBNP, pg/mL, median (IQR)	1745.0 (605.0-5782.0)		
Hemoglobin, g/L^b^	128.9 ± 22.3	132.8 ± 22.7	0.004
Transferrin saturation, %, median (IQR)^c^	20 (14-27)		-
Ferritin, ng/mL, median (IQR)^c^	71.0 (31.5-147.8)		-
Iron deficiency, number (%)^d^	143 (70.8)		-
Creatinine, µmol/L, median (IQR)	91.0 (75.0-115.0)	95.0 (78.0-135.0)	<0.001
eGFR of <60 mL/minute/1.73 m^2^, number (%)	72 (35.6)	93 (46.0)	0.001
eGFR of <30 mL/minute/1.73 m^2^, number (%)	15 (7.4)	29 (14.4)	0.001
Sodium, mmol/L	138.5 ± 3.5	138.3 ± 3.6	0.579
Potassium, mmol/L	4.25 ± 0.53	4.33 ± 0.53	0.028
Hemoglobin A1c, %, median (IQR)	-	6.09 (5.36-7.63)	-
Hemoglobin A1c of >7.0%, number (%)	-	59 (29.2)	-
Low-density lipoprotein, mmol/L, median (IQR)	-	2.1 (1.6-3.1)	-
Atrial fibrillation and/or atrial flutter, number (%)	42 (20.8)		-
Sustained ventricular arrhythmia, number (%)	9 (4.5)		-

There was a numerical decrease in NT-proBNP between the initial and latest visits. Peak median NT-proBNP (1745 pg/mL) was significantly higher compared to baseline values. Overall, 143 (71%) patients met the definition of iron deficiency during follow-up. Severe renal impairment developed in 29 (14%) patients. Potassium increased significantly, and 20 (10%) patients had hyperkalemia at the latest visit.

Data was also analyzed in key subgroups (Table 5). Moderate-to-severe or severe MR was present in 15 (10%) patients with HFrEF versus none in patients without HFrEF (p = 0.025). Patients with a nonischemic cause for HF were a decade younger compared to patients with an ischemic cause of HF (62 versus 51 years, P-value < 0.001). Suboptimally controlled diabetes (44%) and dyslipidemia (53%) remained highly prevalent among patients with an ischemic cause of HF.

**Table 5:** Characteristics of patients and results of investigations in subgroups NT-proBNP, N-terminal pro-B-type natriuretic peptide; LVEF, left ventricular ejection fraction; MR, mitral regurgitation; HBA1c, hemoglobin A1c; LDL, low-density lipoprotein; IQR, interquartile range; HFrEF, heart failure with reduced ejection fraction

	HFrEF	Non-HFrEF	P-value	Ischemic	Nonischemic	P-value
Age, years, median (IQR)	57 (20.8)	51.5 (29)	0.177	63 (16)	51 (21)	<0.001
Female gender, number (%)	51 (33)	16 (33)	1	14 (16)	53 (45)	<0.001
Diabetes, number (%)	83 (54)	20 (42)	0.189	63 (74)	40 (34)	<0.001
Obesity, number (%)	64 (42)	19 (40)	0.94	29 (34)	54 (46)	0.116
Latest NT-proBNP, pg/mL	676 (1592)	243 (550)	<0.001	572 (2708)	525 (1096)	0.165
LVEF, %, median (IQR)	25 (10)	53 (8)	<0.001	28 (13)	33 (28)	<0.001
Moderate-to-severe/severe MR, number (%)	15 (10)	0 (0)	0.025	6 (8)	9 (9)	1
Iron deficiency, number (%)	110 (74)	34 (71)	0.773	63 (79)	81 (70)	0.220
HbA1c of >7.0, number (%)	49 (33)	10 (23)	0.250	35 (44)	24 (22)	0.002
LDL of <1.8 mmol/L, number (%)	52 (34)	16 (35)	1	44 (53)	24 (21)	<0.001

Utilization of medical and ancillary treatments

**Figure 2:**
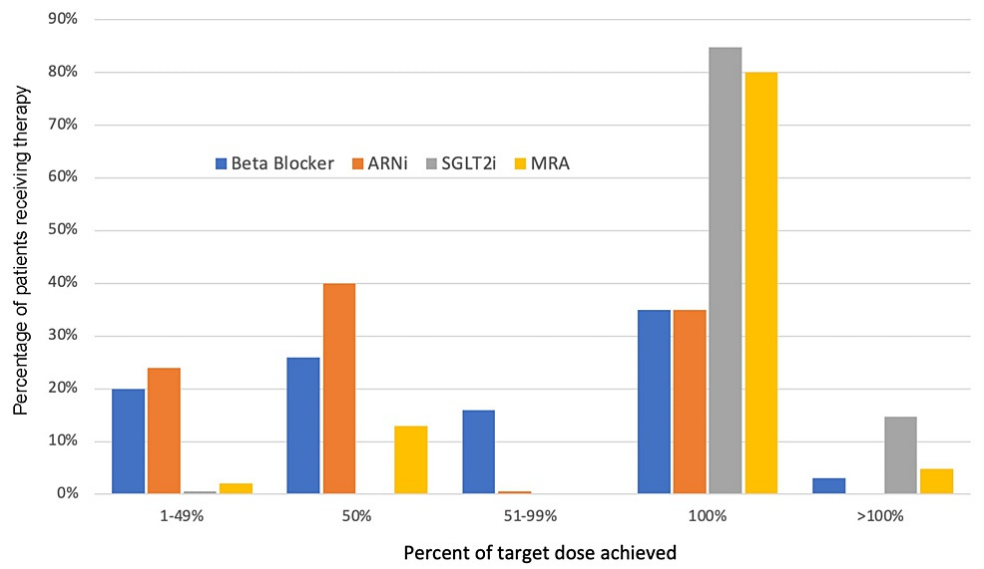
Dosages of key therapies as a proportion of target doses The target daily dose of therapies was in accordance with the guideline recommendations as follows [7]: metoprolol extended-release, 200 mg; bisoprolol, 10 mg; carvedilol, 50 mg; sacubitril-valsartan, 400 mg; empagliflozin, 10 mg; dapagliflozin, 10 mg; and spironolactone, 25 mg ARNi, angiotensin receptor-neprilysin inhibitor; SGLT2i, sodium-glucose cotransporter-2 inhibitor; MRA, mineralocorticoid receptor antagonist

Medical therapy was assessed at the latest visit (Table 6). A majority of patients (88%) were treated with a renin-angiotensin-aldosterone system inhibitor (RAASi). Angiotensin receptor-neprilysin inhibitor (ARNi) was prescribed to 147 (73%) patients, accounting for 83% of the patients with HFrEF. Among those treated with ARNi, the target dose was achieved in 51 (35%) patients (Figure 2). Beta blockers (BB) were the most common treatment. It was prescribed to 196 (97%) patients. Among those treated with BB, the target dose of any BB was achieved in 68 (35%) patients. The SGLT2i and mineralocorticoid receptor antagonists (MRA) were used in 164 (81%) and 148 (73%) patients, respectively. The respective percentages of patients who achieved the target dose of SGLT2i and MRA was 100% and 85%. Overall, 134 (66%) patients were treated with quadruple therapy, consisting of BB, RAASi, SGLT2i, and MRA.

**Table 6:** Treatments and doses of key medications, assessed at the latest visit The distribution of variables is expressed in mean ± standard deviation unless stated otherwise. The following medications were on formulary at the King Faisal Specialist Hospital and Research Centre, Jeddah, and were the most commonly prescribed agents in their respective classes: furosemide, sacubitril-valsartan, lisinopril, losartan, bisoprolol, metoprolol, carvedilol, empagliflozin, and spironolactone. The mean doses were only calculated for the aforementioned medications

Treatment	
Diuretic, number (%)	137 (67.8)
Furosemide dose, mg	68.1 ± 53.1
Angiotensin receptor-neprilysin inhibitor, number (%)	147 (72.8)
Sacubitril-valsartan dose, mg	242.2 ± 119.3
Angiotensin-converting enzyme inhibitor, number (%)	19 (9.4)
Lisinopril, number (%)	15 (7.4)
Others, number (%)	4 (2.0)
Lisinopril dose, mg	6.2 ± 4.6
Angiotensin receptor blocker losartan, number (%)	13 (6.4)
Losartan dose, mg	57.1 ± 35.9
Beta blocker, number (%)	196 (97)
Bisoprolol, number (%)	68 (33.7)
Metoprolol, number (%)	88 (43.6)
Carvedilol, number (%)	36 (17.8)
Bisoprolol dose, mg	6.1 ± 2.9
Metoprolol dose, mg	131.5 ± 56.6
Carvedilol dose, mg	51.0 ± 25.7
Sodium-glucose cotransporter-2 inhibitor, number (%)	164 (81.2)
Empagliflozin, number (%)	162 (80.2)
Empagliflozin dose, mg	12.2 ± 5.4
Mineralocorticoid receptor antagonist, number (%)	148 (73.3)
Spironolactone, number (%)	145 (71.2)
Spironolactone dose, mg	24.5 ± 9.1
Implantable cardiac defibrillator, number (%)	96 (48)
Cardiac resynchronization therapy, number (%)	33 (17)

A novel potassium binder was prescribed to 12 (6%) patients, and an antiarrhythmic agent was prescribed to 15 (7%) patients. Digoxin was prescribed to 34 (17%) patients. Only one patient received ivabradine. Transcatheter edge-to-edge repair was performed in one patient. One patient underwent LVAD implantation, and two patients underwent heart transplantation.

Clinical outcomes

Table 7 presents key outcomes of all patients and patients with and without HFrEF. The composite annual rate of hospitalizations for HF and ED visits for HF was 20%. Patients with HFrEF had a significantly higher composite of HF hospitalizations and ED visits for HF compared to patients without HFrEF (129 versus 20, P-value = 0.01). There were 19 (9%) patients who had at least one HF hospitalization within a year before the study end date despite guideline-directed medical therapy. All these patients were followed for more than one year.

**Table 7:** Outcomes for all patients and a comparison between patients with HFrEF and those without HFrEF ^a^The P-value refers to the comparison between patients with heart failure with reduced ejection fraction (HFrEF) and non-HFrEF. ^b^Mortality was estimated from 200 patients. The status of two patients was unknown

Outcome, number (annual rate, %)	Overall	HFrEF	Non-HFrEF	P-value^a^
Mortality^b^	13 (1.8%)	11 (1.9%)	2 (1.2%)	0.521
Hospitalizations for heart failure (HF)	80 (10.8%)	67 (11.7%)	13 (7.8%)	0.018
Emergency department (ED) visits for HF	69 (9.3%)	62 (10.8%)	7 (4.2%)	0.033
Composite of hospitalizations for HF and ED visits for HF	149 (20.1%)	129 (22.5%)	20 (11.9%)	0.01
Cardiovascular hospitalizations not related to HF	115 (15.5%)	93 (16.2%)	22 (13.1%)	0.162
Non-cardiovascular hospitalizations	77 (10.4%)	54 (9.4%)	23 (13.7%)	0.738

Overall, 13 (6.4%) patients died, which corresponded to an annual rate of mortality of 1.8%. The status of two patients was unknown. Based on a combination of symptoms and NT-proBNP levels, the clinical trajectory of 60% of the patients was adjudicated by the managing physician to be improving (32%) or stable and low risk (28%), whereas the trajectory of 40% was deemed worsening (16%) or high risk and stable (24%).

## Discussion

The present study is a contemporary analysis of the clinical characteristics, management, and outcomes of patients who were followed in a specialized clinic for HF. It raises several salient points that add to the existing literature on the epidemiology of HF in Saudi Arabia.

The average age of this cohort was consistent with previous observations, which demonstrated that patients with HF in Saudi Arabia are younger compared with HF patients elsewhere (Table 8). Although this was previously attributed to the earlier onset of CAD in Saudi Arabia, the age of patients with an ischemic cause of HF in our cohort approximated the age of patients in a landmark trial of ischemic cardiomyopathy [13,14]. More than half of the patients in our cohort had a nonischemic cause for HF, and such patients tend to be younger compared to those with an ischemic cause [15]. The predominance of patients with nonischemic causes likely explained the lower overall age of our cohort. These observations could be attributed to referral bias or an actual change in the local epidemiology of HF. We believe that this warrants further investigation.

**Table 8:** Comparison of key patient characteristics with registries and landmark heart failure trials KFSH&RC-J, King Faisal Specialist Hospital and Research Centre, Jeddah; HEARTS-chronic, Hearts Function Assessment Registry Trial-chronic; INTER-CHF, International Congestive Heart Failure; PARADIGM, Prospective Comparison of ARNi with ACEi to Determine Impact on Global Mortality and Morbidity in Heart Failure; DAPA-HF, Dapagliflozin and Prevention of Adverse Outcomes in Heart Failure; NYHA, New York Heart Association; LVEF, left ventricular ejection fraction; NT-proBNP, N-terminal pro-B-type natriuretic peptide; ACEi, angiotensin-converting enzyme inhibitor; ARB, angiotensin receptor blocker; ARNi, angiotensin receptor-neprilysin inhibitor; MRA, mineralocorticoid receptor antagonists; SGTL2i, sodium-glucose cotransporter-2 inhibitor; ICD, implantable cardiac defibrillator; CRT, cardiac resynchronization therapy; HF, heart failure

	KFSH&RC-J	HEARTS-chronic [5]	INTER-CHF, Middle East [6]	PARADIGM [11]	DAPA-HF [12]
Year	2023	2017	2017	2014	2019
Participants from Saudi Arabia	Yes	Yes	Yes	No	No
Recruited as inpatient, %	0	0	31	0	0
N	202	685	1000	8442	4744
Age, years	55.9	55.7	56	63.8	66.4
Females, %	33	30	28	21.8	23.4
NYHA II-IV, %	76	88	-	94.7	100
Ischemic, %	45	39	50	60	56.4
Body mass index, kg/m^2^	29	28	30	28.2	28.2
LVEF, %	28	-	-	29.5	31.1
Median NT-proBNP (pg/mL)	660	2934	-	1613	1437
Diabetes, %	51	53	57	34.7	41.8
Obese, %	41	-	-	-	-
Atrial fibrillation, %	21	11.5	-	37.4	38.3
Stroke, %	11.4	7	-	8.7	-
Greater than moderate mitral regurgitation, %	7.4	-	-	-	-
Hyperkalemia, %	10	-	-	16.7	-
Iron deficiency, %	71	-	-	-	-
Diuretic, %	68	87	88	80.2	93.5
ACEi/ARB, %	15.8	86	82	50	83.7
ARNi, %	73	0	0	50	10.7
Beta blocker, %	97	94	85	93	96.1
MRA, %	73.3	42	46	55.6	71.1
SGLT2i, %	81	0	0	0	50
ICD, %	48	29.1	-	14.8	26.2
CRT, %	17	8.8	-	6.9	7.5
Death	1.8% annual	9% at one year	9.4% at one year	17%	12.7
HF hospitalization	10.8% annual	-	-	First hospitalization, 14.2	HF hospitalization, 11.6
Urgent visit for HF	9.3% annual	-	-	-	0.7

The majority of patients who were enrolled in the clinic had HFrEF, owing to the low rate of referral for patients with HF with preserved EF (HFpEF). Underdiagnosis and/or a perceived lack of effective therapies for HFpEF could account for their low rate of referral. A significant proportion of patients with nonischemic HF had no identifiable cause, which was consistent with reports that showed that a cause could not be determined in half of subjects with nonischemic cardiomyopathy [7,15].

The two leading comorbid conditions were diabetes and obesity. The prevalence of diabetes in the present study exceeded its prevalence in key HF trials and was similar to previous registries of HF patients from Saudi Arabia. The prevalence of obesity was high overall in the present study (Table 8). These observations may signify the inadequacy of existing mitigation strategies for diabetes and obesity in Saudi Arabia. They highlight the need for a comprehensive approach to address the triad of HF, diabetes, and obesity. Glucagon-like peptide 1 agonists could be poised to play an outsized role should their safety and efficacy be proven across the spectrum of HF.

Dyspnea was the most common symptom, and it improved significantly on follow-up in response to therapy. Patients with stage C HF but without dyspnea at referral had likely responded to treatment commenced prior to the initial visit. Orthostatic dizziness was prevalent, and it conceivably interfered with the maximization of HF medications. Decreases in heart rate and natriuretic peptide (NP) levels and improvements in EF correlated with the overall improvement in symptoms and HF status at the latest visits. The decrease in NP values however was not linear, as evidenced by the substantially elevated peak values during follow-up.

Data on the prevalence of functional MR in patients with HF is limited [16,17]. In the present analysis, approximately 10% of the patients with HFrEF had moderate-to-severe or severe functional MR at the latest visit. Our estimate of the degree of MR could be conservative because transthoracic echocardiography could underestimate the severity of functional MR [18]. Studies that have reported a higher prevalence of severe functional MR included inpatients with acute HF who were prone to having higher grades of MR [16,17]. Although patients in our cohort were well-treated and were potential clinical candidates for mitral transcatheter edge-to-edge repair, additional considerations to qualify for the intervention include the anatomic suitability of the mitral valve, LVEF, and LV size [7].

The prevalence of iron deficiency in HF patients in Saudi Arabia is unknown. More than 70% of the patients in our cohort were iron-deficient, a prevalence that was higher than the 50%-60% prevalence reported globally [19,20]. This emphasizes the need for resource allocation to treat iron deficiency in HF patients per the guidelines [7,8]. The lack of differences in iron test results among relevant subgroups suggested that non-HF contributors to iron deficiency were less likely. Because iron deficiency is more common in acute HF, we could not exclude an interaction between the iron profile and HF status.

Moderate or severe chronic kidney disease (CKD) was highly prevalent, portending a poorer prognosis and the suboptimal use of HF therapies [21,22]. The prevalence of hyperkalemia after the treatment of HF suggested that 10% of the patients could meet the indication for novel potassium binders.

In the present analysis, 2/3 of patients were treated with quadruple therapy. The SGLT2i was more likely to be used in comparison to ARNi, reflecting their broader indications and ease of use. The use of MRA exceeded that in landmark trials of HFrEF and previous registries (Table 8). Only 35% of the patients achieved the maximum dose of ARNi or BB despite a follow-up of at least six months. By comparison, 76% of the patients reached and maintained the target dose of ARNi within three months of initiation in a clinical trial of dose titration of ARNi [23]. In another study, 65% of the patients reached the target dose of ARNi by one year [24]. With respect to BB, 65% of the patients reached the target dose of carvedilol within four months of initiation in a clinical trial of patients with severe HF [25]. On the other hand, target doses of SGLT2i and MRA in our study were achieved in more than 80% of the patients. These observations highlight that allocating resources that ensure frequent follow-up could help overcome barriers in up-titrating ARNi and BB and achieve better outcomes [26].

All-cause mortality in the present study was lower in comparison to previous reports, which could be explained by the selection of higher-risk patients in those studies (Table 8). Landmark clinical trials of HF were designed to recruit outpatients at high risk of events, by selecting patients who were symptomatic and had elevated levels of NT-proBNP [11,12]. The Hearts Function Assessment Registry Trial-chronic (HEARTS-chronic) was one of the registries that included patients with HF in Saudi Arabia and specifically recruited outpatients with severe symptoms and LV dysfunction that were at high risk of hospitalization [5]. The International Congestive Heart Failure (INTER-CHF) was another study that included patients with HF from Saudi Arabia and recruited 30% of the patients during hospitalization for HF [6]. In our relatively unselected cohort of patients, 1/4 had no dyspnea at the initial visit. The lower rate of use of diuretics, the lower NP level, and the increase in EF indicated that the overall risk profile was low and/or improved on follow-up. Some of these favorable effects on mortality could be because of the effectiveness of modern HF therapy. All deaths occurred among patients who were deemed to have a high-risk or worsening HF trajectory. Nevertheless, the annual rates of HF hospitalizations and ED visits for HF represented a significant burden. Approximately 19 (9%) patients who had at least one hospitalization within the year prior to the study end date despite guideline-directed medical therapy for >1 year were potential candidates for the implantation of a pulmonary artery pressure monitor.

Limitations

The present study is a single-center study with a small sample size that could limit its generalizability. However, many features of our patients were aligned with previous reports of patients with HF, and our cohort was relatively unselected to reflect real-world practice patterns in Saudi Arabia. Because the exclusion of patients with a follow-up of <6 months could have excluded patients with rapid disease progression, we included those who died within six months of enrolment in the clinic. The retrospective nature of the study risked deficiencies and inaccuracies in data. However, standardized documentation in the HF Clinic and the implementation of electronic charting since the inception of the clinic contributed to the integrity of the data. Clinical events other than deaths may not have been captured for patients residing in distant locations because they could seek medical care at their local facilities. Our estimates with respect to those events were therefore conservative.

## Conclusions

This contemporary cohort of outpatients with HF was relatively young due to the predominance of patients with nonischemic causes of HF. We reported a strikingly high prevalence of diabetes, obesity, and iron deficiency. Although the uptake of quadruple therapy was high, achieving target doses remained a challenge. This was the first known regional analysis to estimate the proportion of potential candidates for intravenous iron replacement, transcatheter repair of the mitral valve, novel potassium binders, and implantable pulmonary artery pressure monitor. The low mortality of the cohort may have reflected the risk profile of patients enrolled in the clinic and the effect of modern therapies, although the burden of HF events remained elevated. This study provided relevant benchmarks for resource planning and future investigations.
